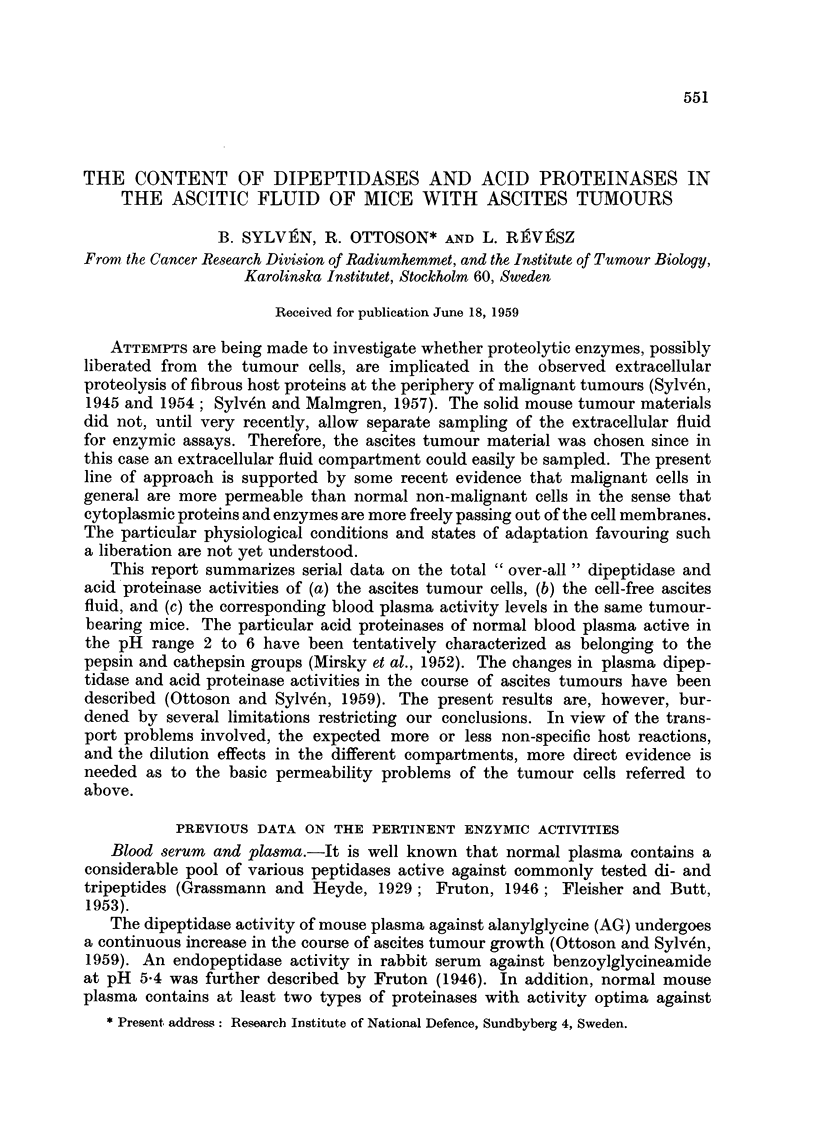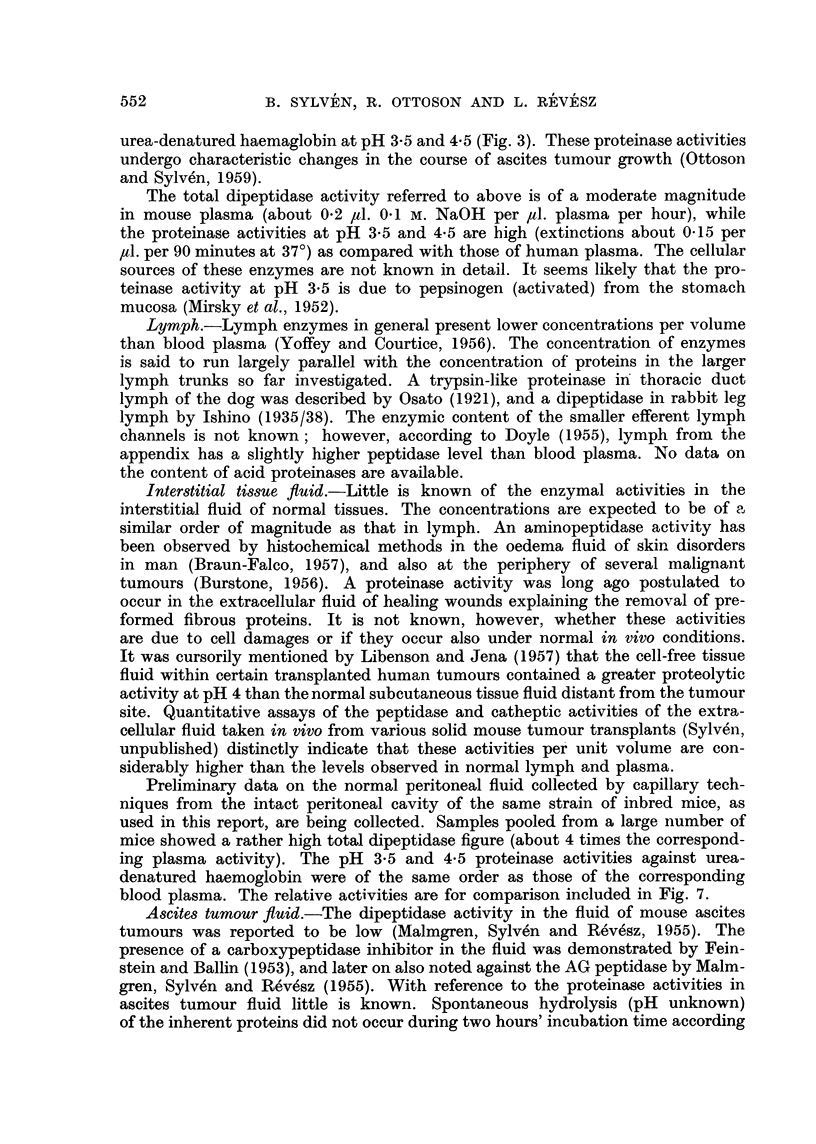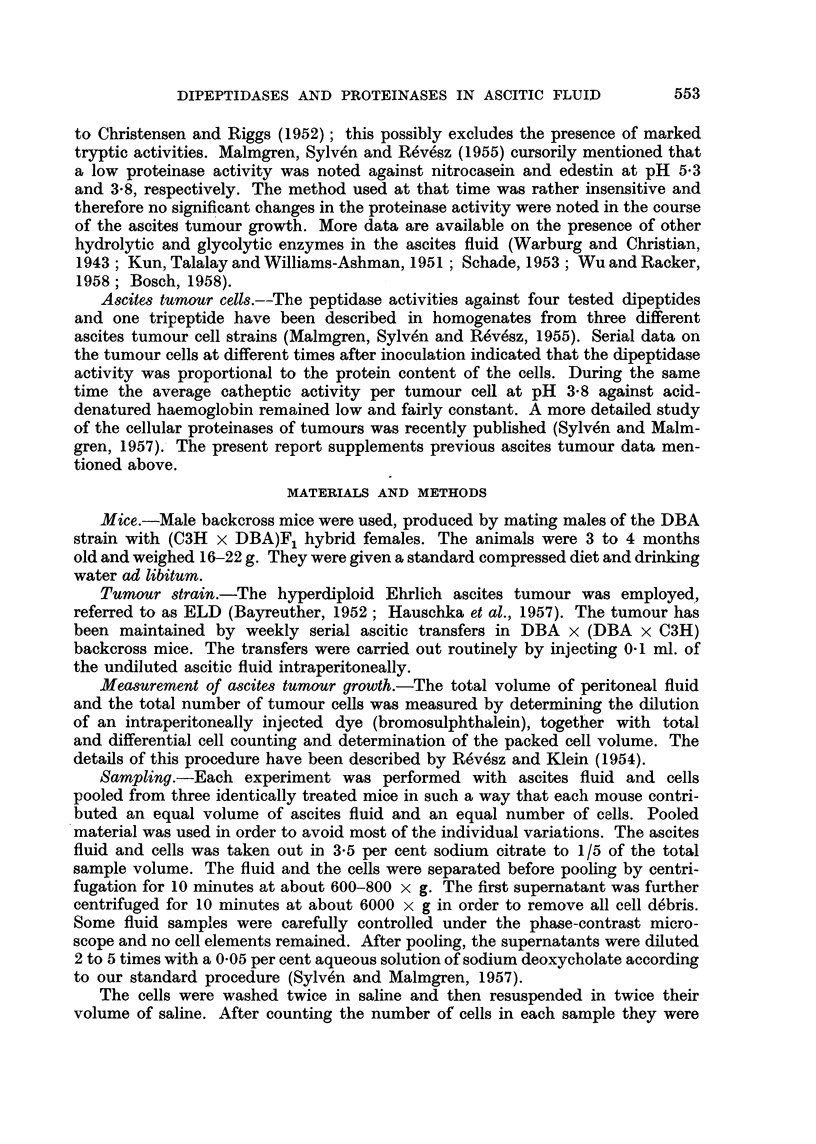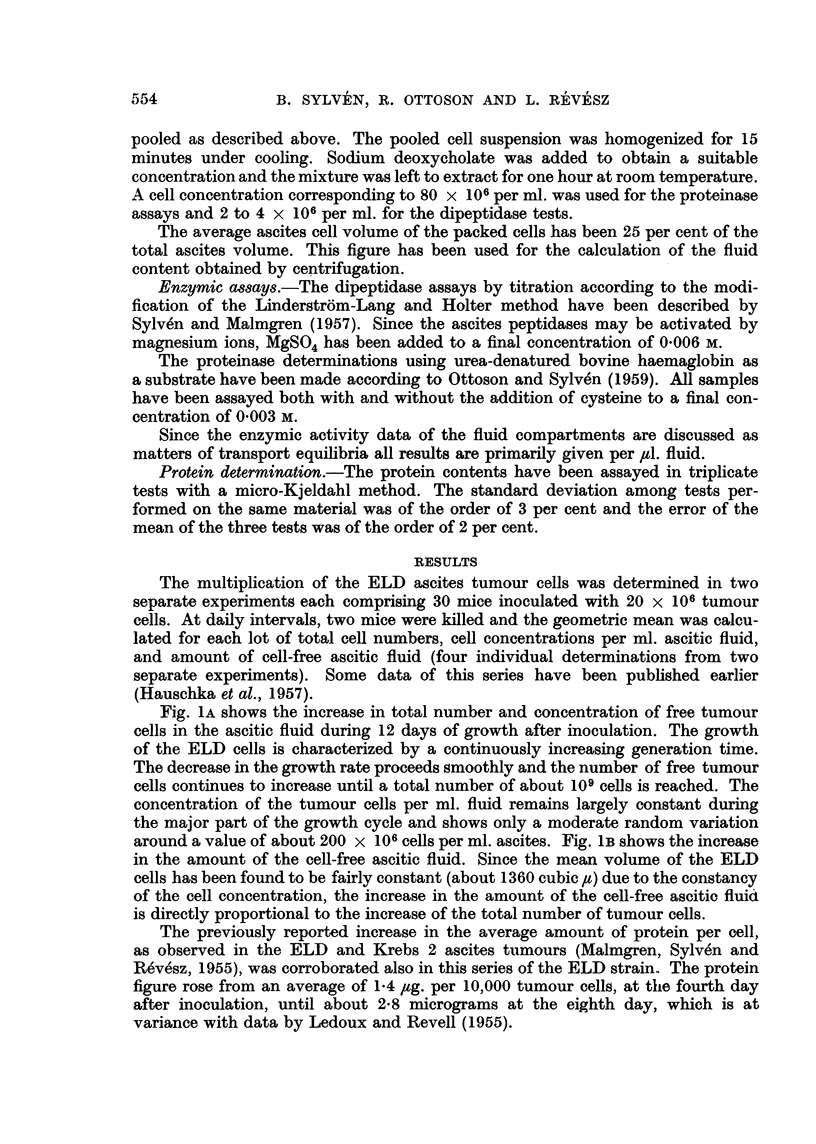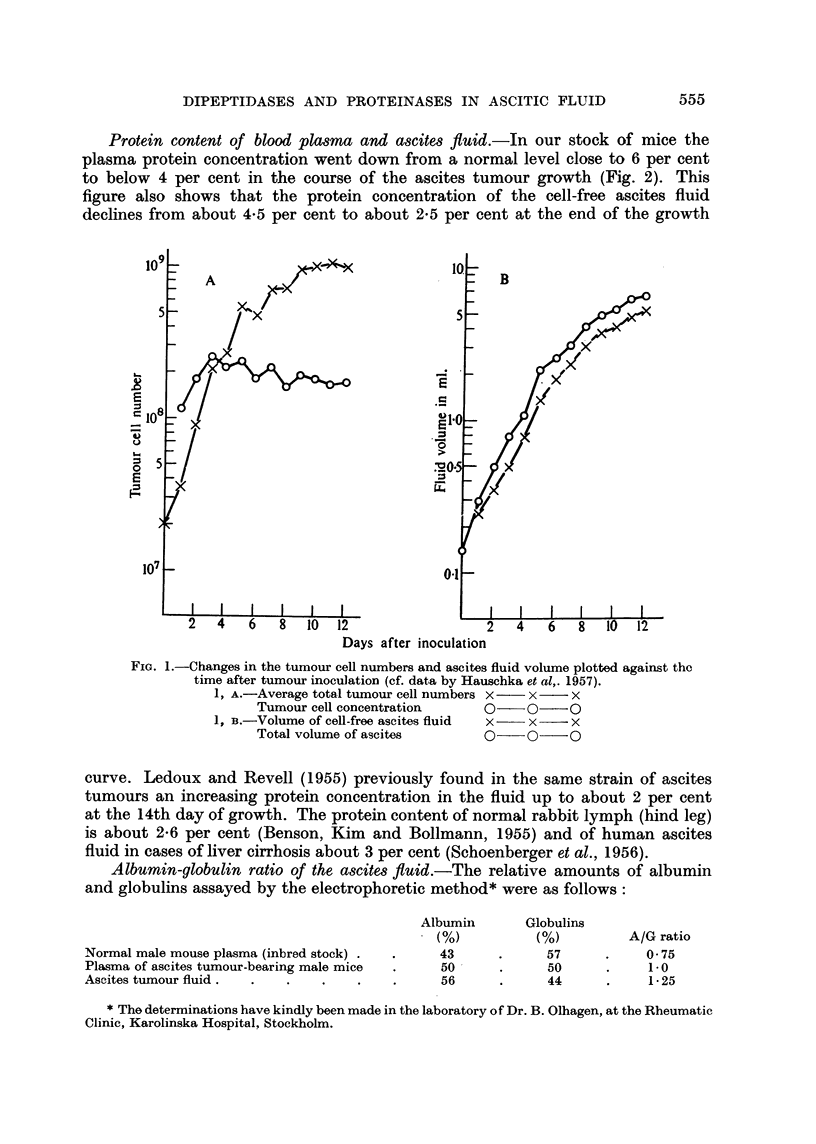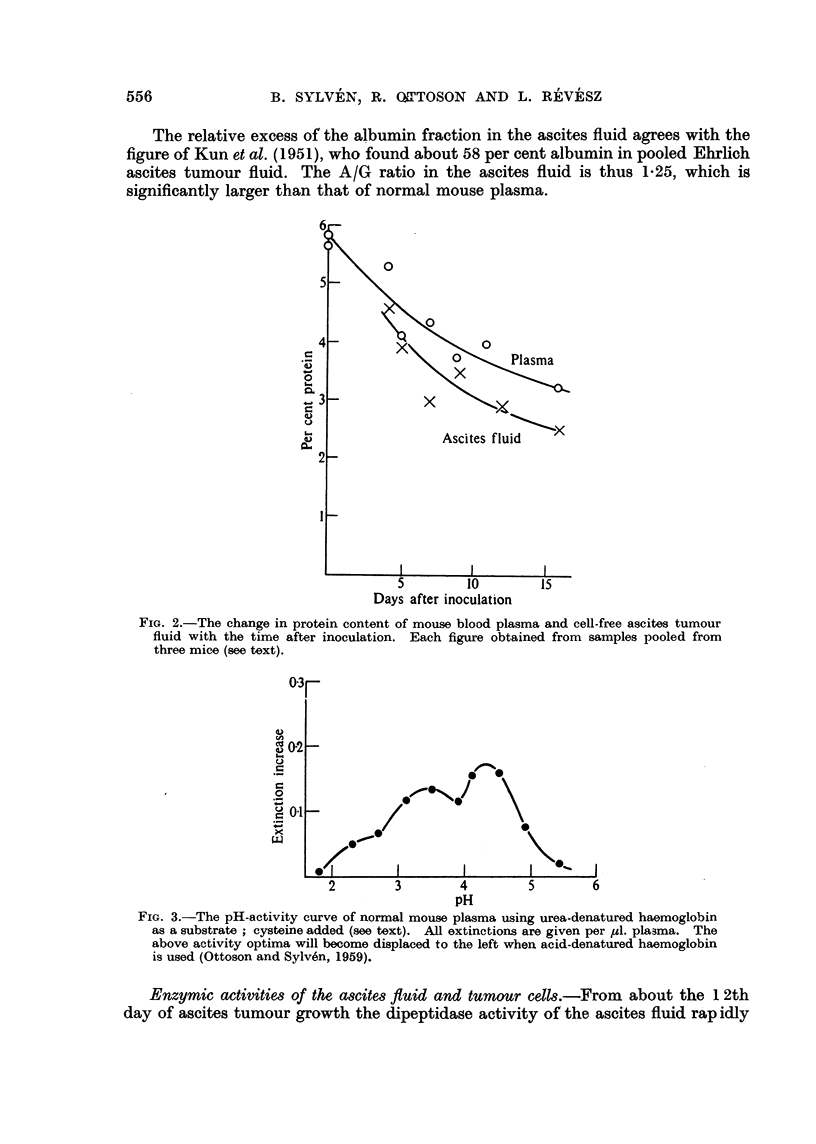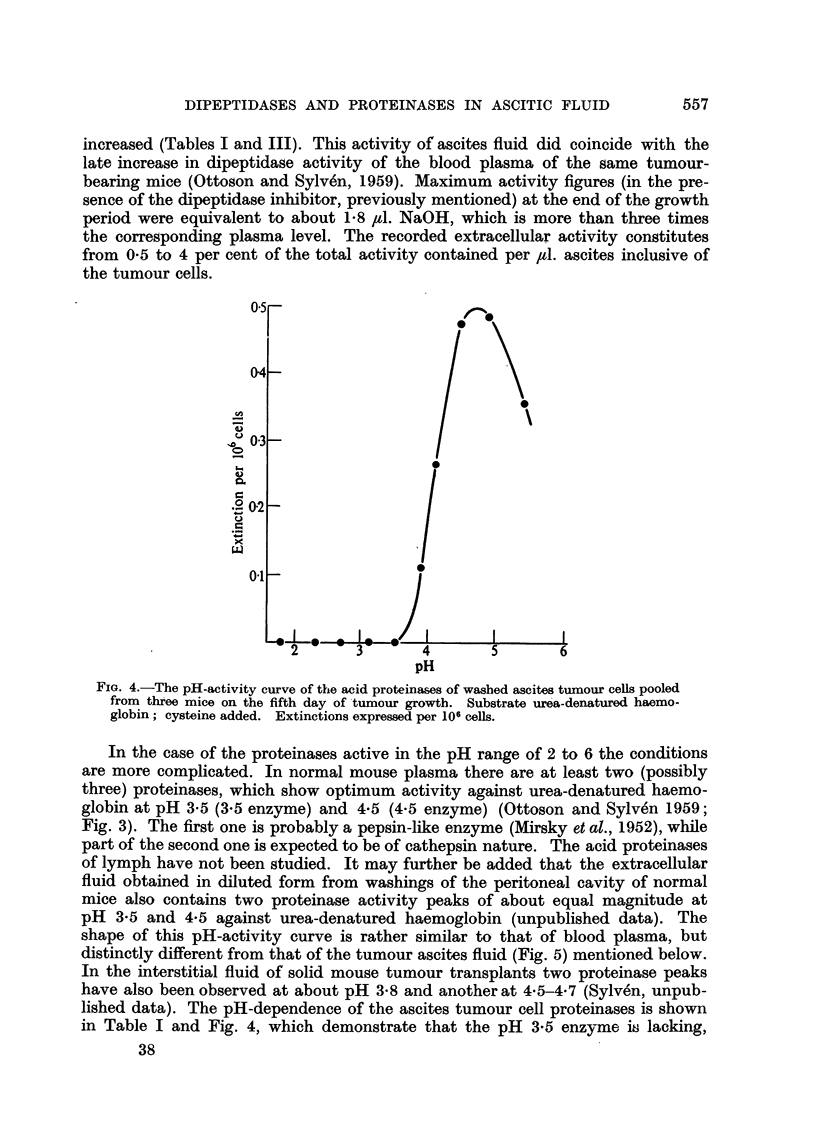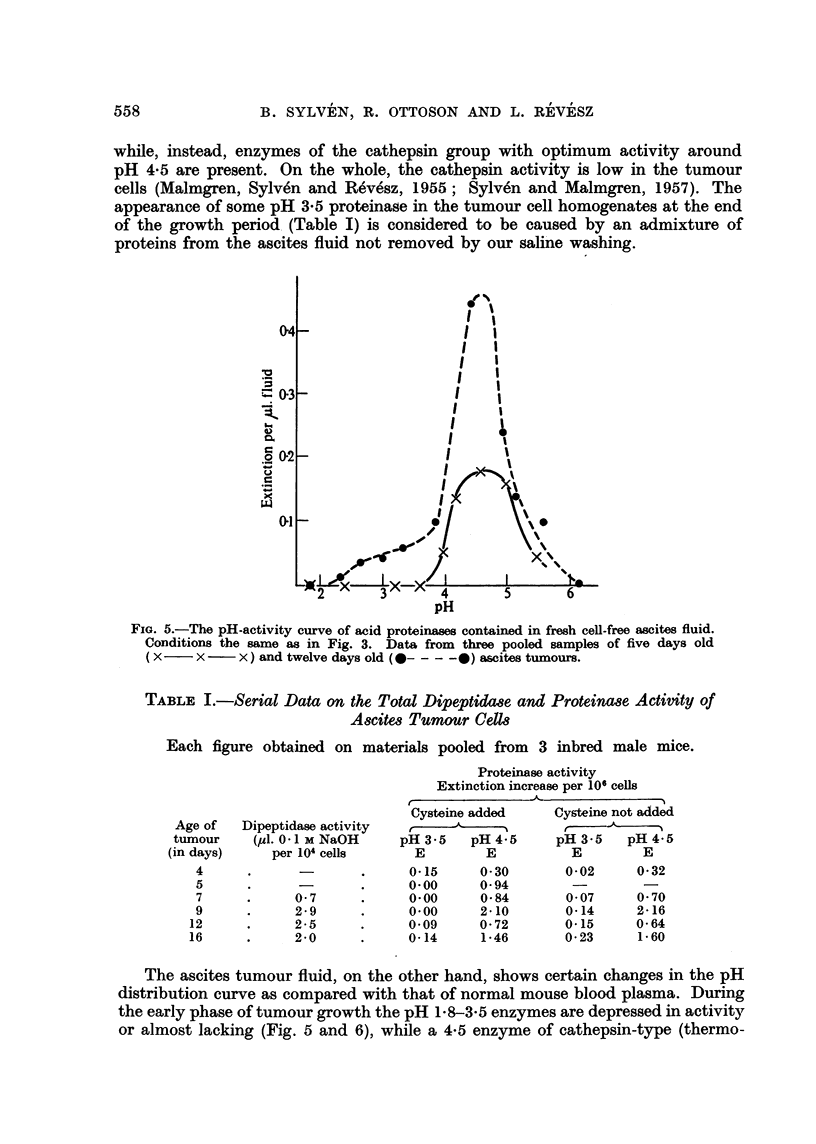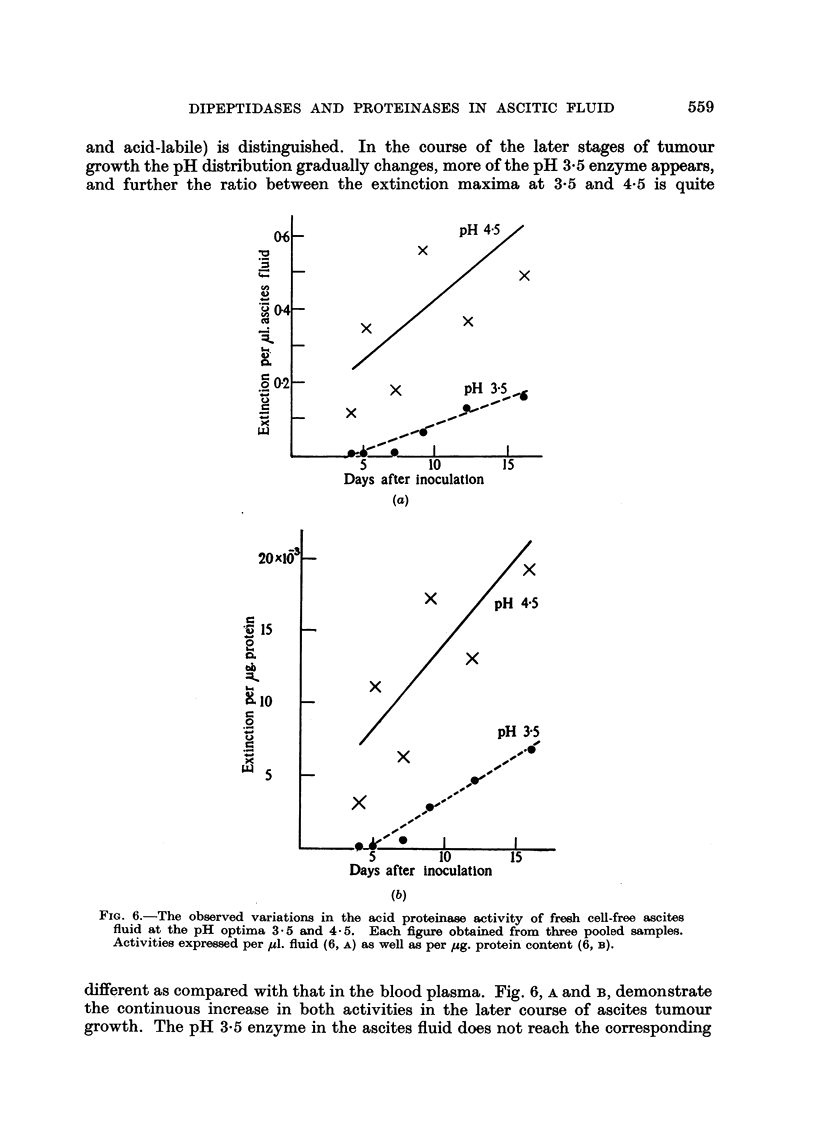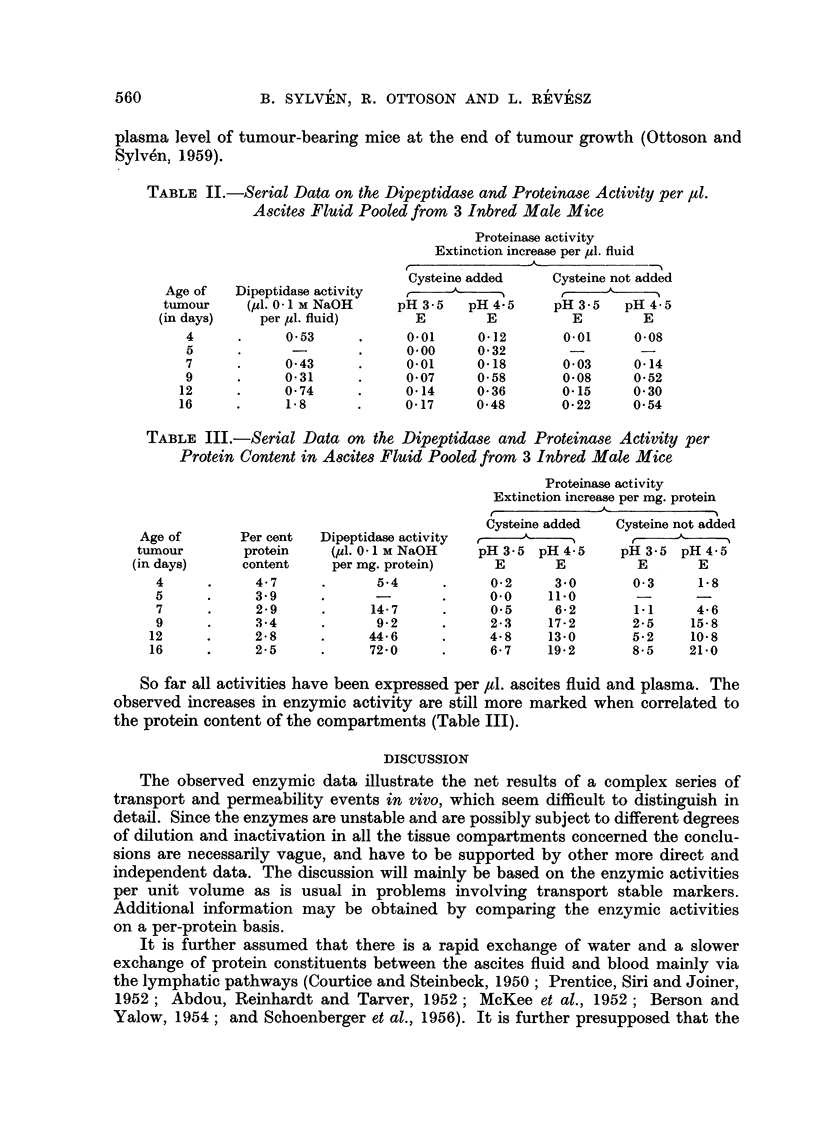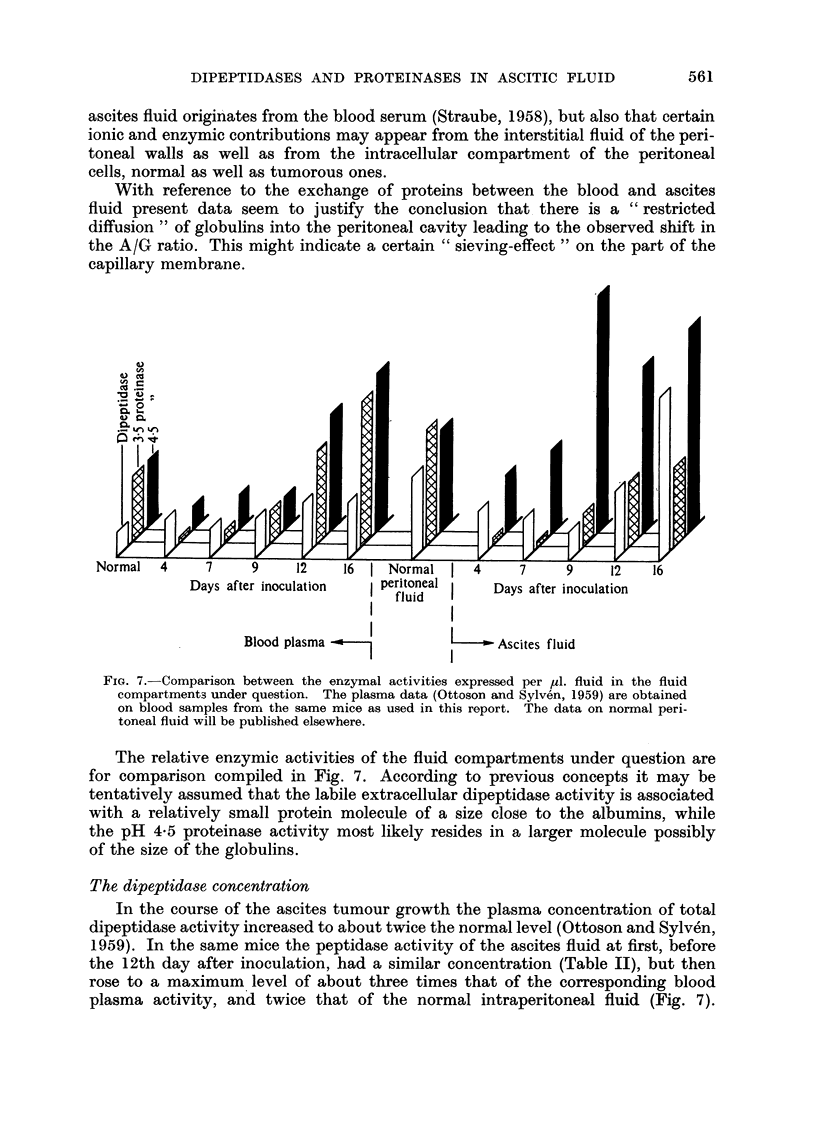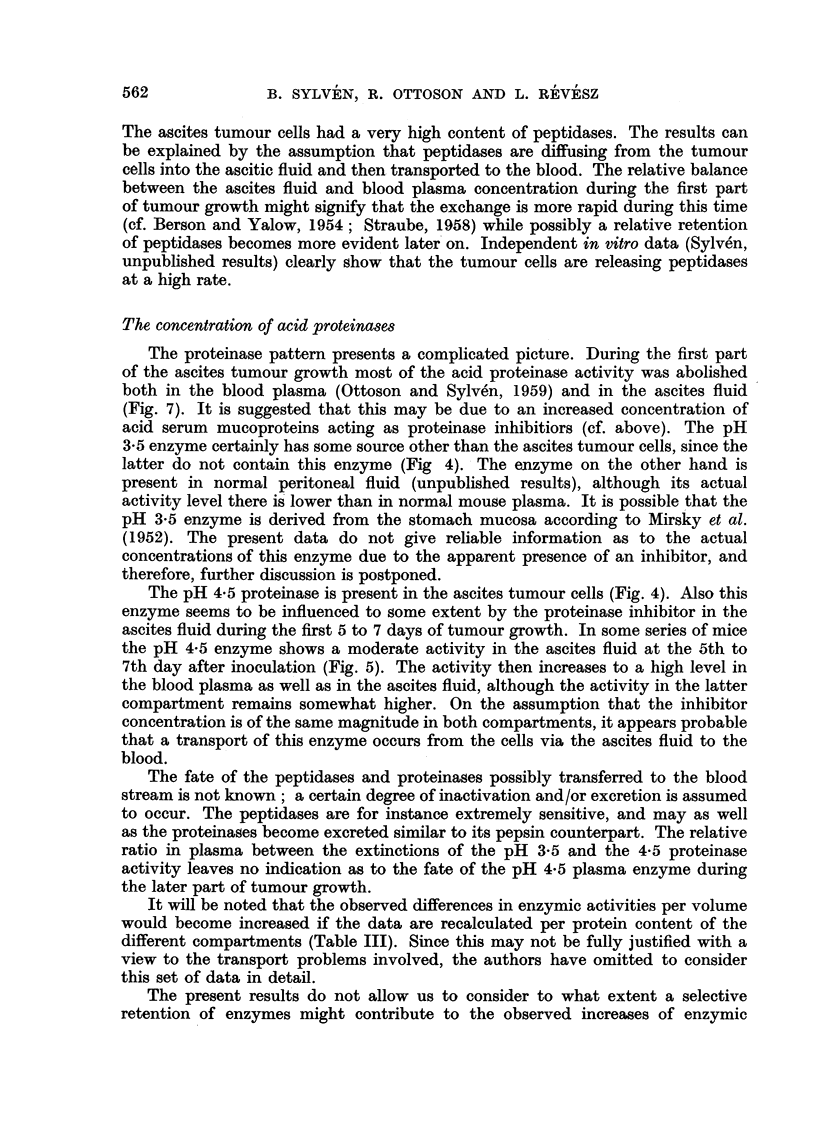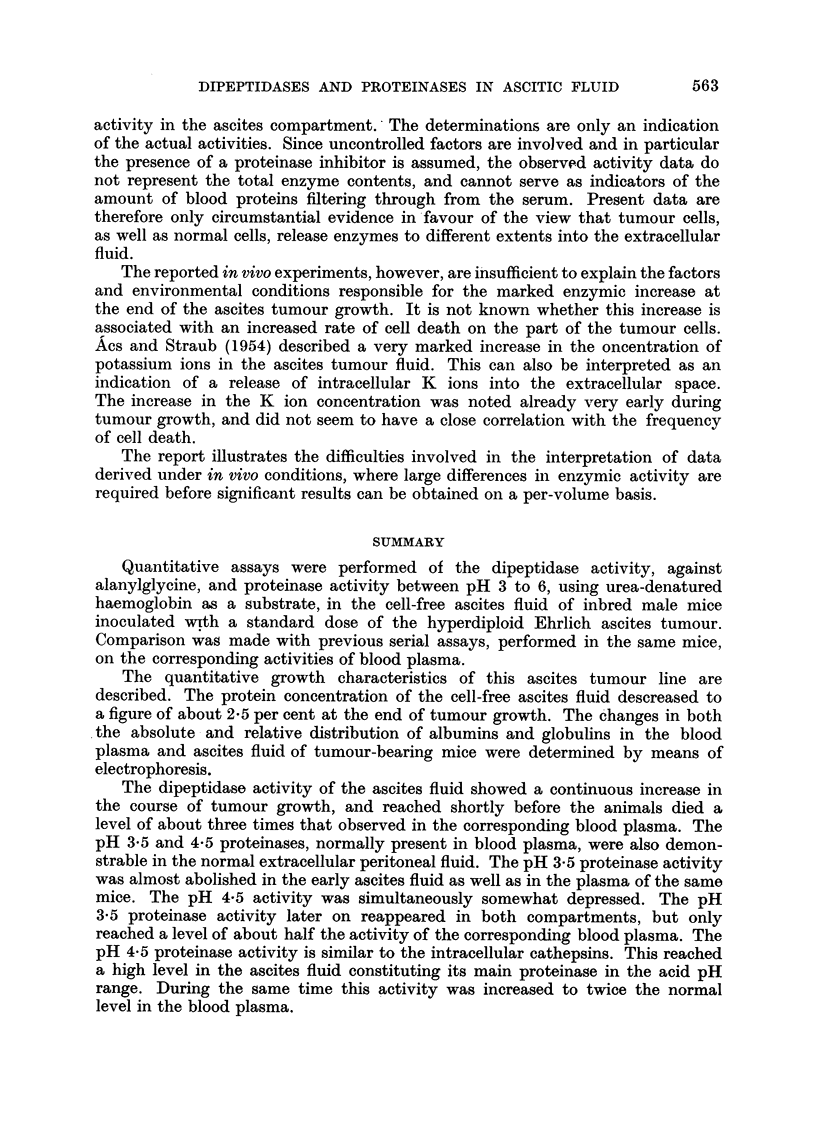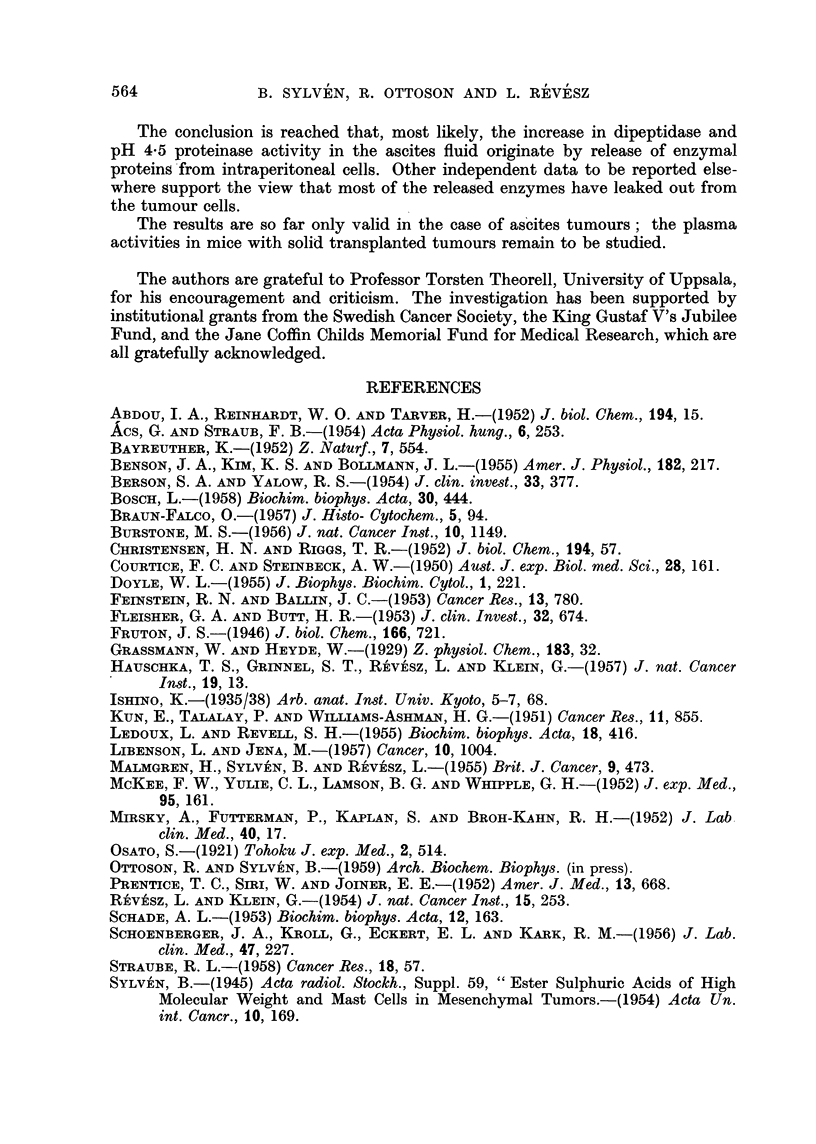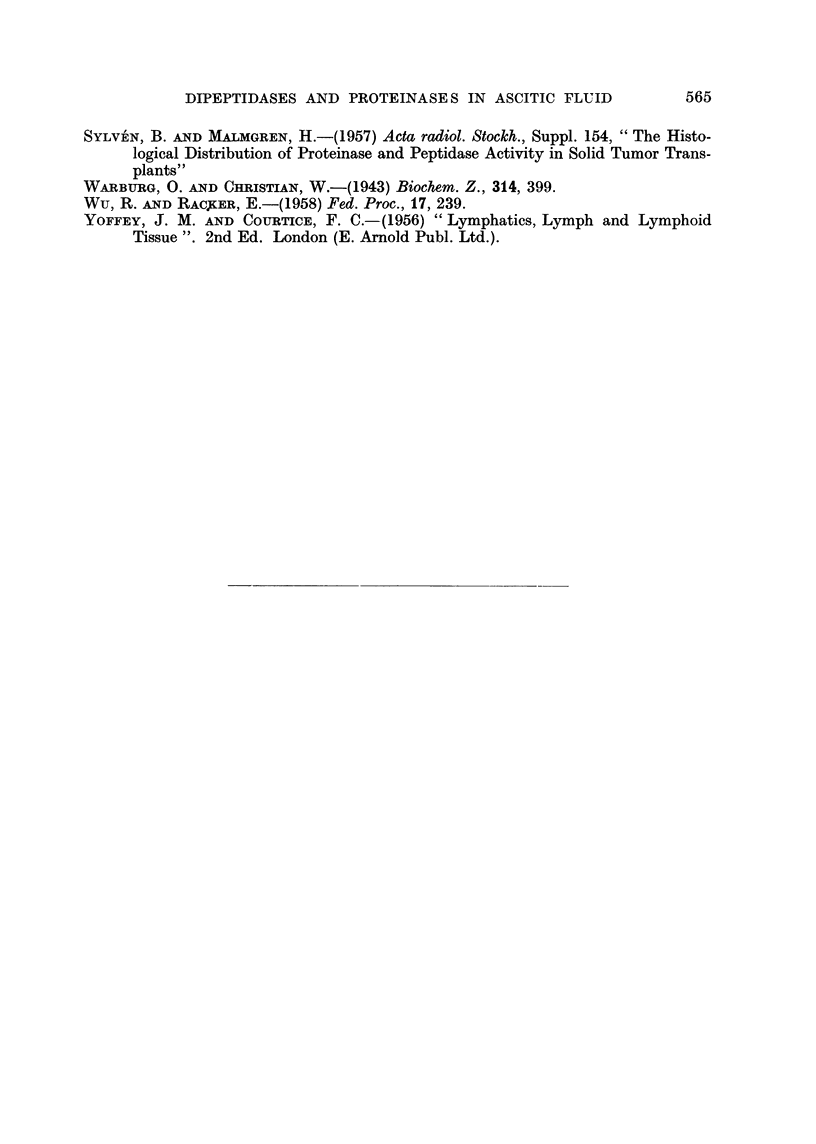# The Content of Dipeptidases and Acid Proteinases in the Ascitic Fluid of Mice with Ascites Tumours

**DOI:** 10.1038/bjc.1959.60

**Published:** 1959-09

**Authors:** B. Sylvén, R. Ottoson, L. Révész


### THE CONTENT OF DIPEPTIDASES AND ACID PROTEINASES IN THE ASCITIC FLUID OF MICE WITH ASCITES TUMOURS

B. SYLVÉN, R. OTTOSON* AND L. RÉVÉSZ

*From the Cancer Research Division of Radiumhemmet, and the Institute of Tumour Biology, Karolinska Institutet, Stockholm 60, Sweden*

Received for publication June 18, 1959

ATTEMPTS are being made to investigate whether proteolytic enzymes, possibly liberated from the tumour cells, are implicated in the observed extracellular proteolysis of fibrous host proteins at the periphery of malignant tumours (Sylvén, 1945 and 1954 ; Sylvén and Malmgren, 1957). The solid mouse tumour materials did not, until very recently, allow separate sampling of the extracellular fluid for enzymic assays. Therefore, the ascites tumour material was chosen since in this case an extracellular fluid compartment could easily be sampled. The present line of approach is supported by some recent evidence that malignant cells in general are more permeable than normal non-malignant cells in the sense that cytoplasmic proteins and enzymes are more freely passing out of the cell membranes. The particular physiological conditions and states of adaptation favouring such a liberation are not yet understood.

This report summarizes serial data on the total " over-all " dipeptidase and acid proteinase activities of (*a*) the ascites tumour cells, (*b*) the cell-free ascites fluid, and (*c*) the corresponding blood plasma activity levels in the same tumour-bearing mice. The particular acid proteinases of normal blood plasma active in the pH range 2 to 6 have been tentatively characterized as belonging to the pepsin and cathepsin groups (Mirsky *et al.*, 1952). The changes in plasma dipeptidase and acid proteinase activities in the course of ascites tumours have been described (Ottoson and Sylvén, 1959). The present results are, however, burdened by several limitations restricting our conclusions. In view of the transport problems involved, the expected more or less non-specific host reactions, and the dilution effects in the different compartments, more direct evidence is needed as to the basic permeability problems of the tumour cells referred to above.

### PREVIOUS DATA ON THE PERTINENT ENZYMIC ACTIVITIES

*Blood serum and plasma.*—It is well known that normal plasma contains a considerable pool of various peptidases active against commonly tested di- and tripeptides (Grassmann and Heyde, 1929 ; Fruton, 1946 ; Fleisher and Butt, 1953).

The dipeptidase activity of mouse plasma against alanylglycine (AG) undergoes a continuous increase in the course of ascites tumour growth (Ottoson and Sylvén, 1959). An endopeptidase activity in rabbit serum against benzoylglycineamide at pH 5·4 was further described by Fruton (1946). In addition, normal mouse plasma contains at least two types of proteinases with activity optima against

* Present address : Research Institute of National Defence, Sundbyberg 4, Sweden.

urea-denatured haemaglobin at pH 3·5 and 4·5 (Fig. 3). These proteinase activities undergo characteristic changes in the course of ascites tumour growth (Ottoson and Sylvén, 1959).

The total dipeptidase activity referred to above is of a moderate magnitude in mouse plasma (about 0·2 $\mu$l. 0·1 M. NaOH per $\mu$l. plasma per hour), while the proteinase activities at pH 3·5 and 4·5 are high (extinctions about 0·15 per $\mu$l. per 90 minutes at 37°) as compared with those of human plasma. The cellular sources of these enzymes are not known in detail. It seems likely that the proteinase activity at pH 3·5 is due to pepsinogen (activated) from the stomach mucosa (Mirsky *et al.*, 1952).

*Lymph.*—Lymph enzymes in general present lower concentrations per volume than blood plasma (Yoffey and Courtice, 1956). The concentration of enzymes is said to run largely parallel with the concentration of proteins in the larger lymph trunks so far investigated. A trypsin-like proteinase in thoracic duct lymph of the dog was described by Osato (1921), and a dipeptidase in rabbit leg lymph by Ishino (1935/38). The enzymic content of the smaller efferent lymph channels is not known ; however, according to Doyle (1955), lymph from the appendix has a slightly higher peptidase level than blood plasma. No data on the content of acid proteinases are available.

*Interstitial tissue fluid.*—Little is known of the enzymal activities in the interstitial fluid of normal tissues. The concentrations are expected to be of a similar order of magnitude as that in lymph. An aminopeptidase activity has been observed by histochemical methods in the oedema fluid of skin disorders in man (Braun-Falco, 1957), and also at the periphery of several malignant tumours (Burstone, 1956). A proteinase activity was long ago postulated to occur in the extracellular fluid of healing wounds explaining the removal of preformed fibrous proteins. It is not known, however, whether these activities are due to cell damages or if they occur also under normal *in vivo* conditions. It was cursorily mentioned by Libenson and Jena (1957) that the cell-free tissue fluid within certain transplanted human tumours contained a greater proteolytic activity at pH 4 than the normal subcutaneous tissue fluid distant from the tumour site. Quantitative assays of the peptidase and catheptic activities of the extracellular fluid taken *in vivo* from various solid mouse tumour transplants (Sylvén, unpublished) distinctly indicate that these activities per unit volume are considerably higher than the levels observed in normal lymph and plasma.

Preliminary data on the normal peritoneal fluid collected by capillary techniques from the intact peritoneal cavity of the same strain of inbred mice, as used in this report, are being collected. Samples pooled from a large number of mice showed a rather high total dipeptidase figure (about 4 times the corresponding plasma activity). The pH 3·5 and 4·5 proteinase activities against urea-denatured haemoglobin were of the same order as those of the corresponding blood plasma. The relative activities are for comparison included in Fig. 7.

*Ascites tumour fluid.*—The dipeptidase activity in the fluid of mouse ascites tumours was reported to be low (Malmgren, Sylvén and Révész, 1955). The presence of a carboxypeptidase inhibitor in the fluid was demonstrated by Feinstein and Ballin (1953), and later on also noted against the AG peptidase by Malmgren, Sylvén and Révész (1955). With reference to the proteinase activities in ascites tumour fluid little is known. Spontaneous hydrolysis (pH unknown) of the inherent proteins did not occur during two hours' incubation time according to Christensen and Riggs (1952); this possibly excludes the presence of marked tryptic activities. Malmgren, Sylvén and Révész (1955) cursorily mentioned that a low proteinase activity was noted against nitrocasein and edestin at pH 5·3 and 3·8, respectively. The method used at that time was rather insensitive and therefore no significant changes in the proteinase activity were noted in the course of the ascites tumour growth. More data are available on the presence of other hydrolytic and glycolytic enzymes in the ascites fluid (Warburg and Christian, 1943; Kun, Talalay and Williams-Ashman, 1951; Schade, 1953; Wu and Racker, 1958; Bosch, 1958).

*Ascites tumour cells.*—The peptidase activities against four tested dipeptides and one tripeptide have been described in homogenates from three different ascites tumour cell strains (Malmgren, Sylvén and Révész, 1955). Serial data on the tumour cells at different times after inoculation indicated that the dipeptidase activity was proportional to the protein content of the cells. During the same time the average catheptic activity per tumour cell at pH 3·8 against acid-denatured haemoglobin remained low and fairly constant. A more detailed study of the cellular proteinases of tumours was recently published (Sylvén and Malmgren, 1957). The present report supplements previous ascites tumour data mentioned above.

### MATERIALS AND METHODS

*Mice.*—Male backcross mice were used, produced by mating males of the DBA strain with (C3H × DBA)$F_1$ hybrid females. The animals were 3 to 4 months old and weighed 16–22 g. They were given a standard compressed diet and drinking water *ad libitum.*

*Tumour strain.*—The hyperdiploid Ehrlich ascites tumour was employed, referred to as ELD (Bayreuther, 1952; Hauschka *et al.*, 1957). The tumour has been maintained by weekly serial ascitic transfers in DBA × (DBA × C3H) backcross mice. The transfers were carried out routinely by injecting 0·1 ml. of the undiluted ascitic fluid intraperitoneally.

*Measurement of ascites tumour growth.*—The total volume of peritoneal fluid and the total number of tumour cells was measured by determining the dilution of an intraperitoneally injected dye (bromosulphthalein), together with total and differential cell counting and determination of the packed cell volume. The details of this procedure have been described by Révész and Klein (1954).

*Sampling.*—Each experiment was performed with ascites fluid and cells pooled from three identically treated mice in such a way that each mouse contributed an equal volume of ascites fluid and an equal number of cells. Pooled material was used in order to avoid most of the individual variations. The ascites fluid and cells was taken out in 3·5 per cent sodium citrate to 1/5 of the total sample volume. The fluid and the cells were separated before pooling by centrifugation for 10 minutes at about 600–800 × g. The first supernatant was further centrifuged for 10 minutes at about 6000 × g in order to remove all cell débris. Some fluid samples were carefully controlled under the phase-contrast microscope and no cell elements remained. After pooling, the supernatants were diluted 2 to 5 times with a 0·05 per cent aqueous solution of sodium deoxycholate according to our standard procedure (Sylvén and Malmgren, 1957).

The cells were washed twice in saline and then resuspended in twice their volume of saline. After counting the number of cells in each sample they were pooled as described above. The pooled cell suspension was homogenized for 15 minutes under cooling. Sodium deoxycholate was added to obtain a suitable concentration and the mixture was left to extract for one hour at room temperature. A cell concentration corresponding to 80 $\times$ $10^6$ per ml. was used for the proteinase assays and 2 to 4 $\times$ $10^6$ per ml. for the dipeptidase tests.

The average ascites cell volume of the packed cells has been 25 per cent of the total ascites volume. This figure has been used for the calculation of the fluid content obtained by centrifugation.

*Enzymic assays.*—The dipeptidase assays by titration according to the modification of the Linderström-Lang and Holter method have been described by Sylvén and Malmgren (1957). Since the ascites peptidases may be activated by magnesium ions, $MgSO_4$ has been added to a final concentration of 0·006 M.

The proteinase determinations using urea-denatured bovine haemaglobin as a substrate have been made according to Ottoson and Sylvén (1959). All samples have been assayed both with and without the addition of cysteine to a final concentration of 0·003 M.

Since the enzymic activity data of the fluid compartments are discussed as matters of transport equilibria all results are primarily given per $\mu$l. fluid.

*Protein determination.*—The protein contents have been assayed in triplicate tests with a micro-Kjeldahl method. The standard deviation among tests performed on the same material was of the order of 3 per cent and the error of the mean of the three tests was of the order of 2 per cent.

### RESULTS

The multiplication of the ELD ascites tumour cells was determined in two separate experiments each comprising 30 mice inoculated with 20 $\times$ $10^6$ tumour cells. At daily intervals, two mice were killed and the geometric mean was calculated for each lot of total cell numbers, cell concentrations per ml. ascitic fluid, and amount of cell-free ascitic fluid (four individual determinations from two separate experiments). Some data of this series have been published earlier (Hauschka *et al.*, 1957).

Fig. 1A shows the increase in total number and concentration of free tumour cells in the ascitic fluid during 12 days of growth after inoculation. The growth of the ELD cells is characterized by a continuously increasing generation time. The decrease in the growth rate proceeds smoothly and the number of free tumour cells continues to increase until a total number of about $10^9$ cells is reached. The concentration of the tumour cells per ml. fluid remains largely constant during the major part of the growth cycle and shows only a moderate random variation around a value of about 200 $\times$ $10^6$ cells per ml. ascites. Fig. 1B shows the increase in the amount of the cell-free ascitic fluid. Since the mean volume of the ELD cells has been found to be fairly constant (about 1360 cubic $\mu$) due to the constancy of the cell concentration, the increase in the amount of the cell-free ascitic fluid is directly proportional to the increase of the total number of tumour cells.

The previously reported increase in the average amount of protein per cell, as observed in the ELD and Krebs 2 ascites tumours (Malmgren, Sylvén and Révész, 1955), was corroborated also in this series of the ELD strain. The protein figure rose from an average of 1·4 $\mu$g. per 10,000 tumour cells, at the fourth day after inoculation, until about 2·8 micrograms at the eighth day, which is at variance with data by Ledoux and Revell (1955).

*Protein content of blood plasma and ascites fluid.*—In our stock of mice the plasma protein concentration went down from a normal level close to 6 per cent to below 4 per cent in the course of the ascites tumour growth (Fig. 2). This figure also shows that the protein concentration of the cell-free ascites fluid declines from about 4·5 per cent to about 2·5 per cent at the end of the growth

FIG. 1.—Changes in the tumour cell numbers and ascites fluid volume plotted against the time after tumour inoculation (cf. data by Hauschka *et al*,. 1957).
1, A.—Average total tumour cell numbers ×——×——×
Tumour cell concentration ○——○——○
1, B.—Volume of cell-free ascites fluid ×——×——×
Total volume of ascites ○——○——○

curve. Ledoux and Revell (1955) previously found in the same strain of ascites tumours an increasing protein concentration in the fluid up to about 2 per cent at the 14th day of growth. The protein content of normal rabbit lymph (hind leg) is about 2·6 per cent (Benson, Kim and Bollmann, 1955) and of human ascites fluid in cases of liver cirrhosis about 3 per cent (Schoenberger *et al.*, 1956).

*Albumin-globulin ratio of the ascites fluid.*—The relative amounts of albumin and globulins assayed by the electrophoretic method* were as follows :

| | Albumin (%) | Globulins (%) | A/G ratio |
|---|---|---|---|
| Normal male mouse plasma (inbred stock) | 43 | 57 | 0·75 |
| Plasma of ascites tumour-bearing male mice | 50 | 50 | 1·0 |
| Ascites tumour fluid | 56 | 44 | 1·25 |

* The determinations have kindly been made in the laboratory of Dr. B. Olhagen, at the Rheumatic Clinic, Karolinska Hospital, Stockholm.

The relative excess of the albumin fraction in the ascites fluid agrees with the figure of Kun *et al.* (1951), who found about 58 per cent albumin in pooled Ehrlich ascites tumour fluid. The A/G ratio in the ascites fluid is thus 1·25, which is significantly larger than that of normal mouse plasma.

FIG. 2.—The change in protein content of mouse blood plasma and cell-free ascites tumour fluid with the time after inoculation. Each figure obtained from samples pooled from three mice (see text).

FIG. 3.—The pH-activity curve of normal mouse plasma using urea-denatured haemoglobin as a substrate ; cysteine added (see text). All extinctions are given per $\mu$l. plasma. The above activity optima will become displaced to the left when acid-denatured haemoglobin is used (Ottoson and Sylvén, 1959).

*Enzymic activities of the ascites fluid and tumour cells.*—From about the 12th day of ascites tumour growth the dipeptidase activity of the ascites fluid rapidly increased (Tables I and III). This activity of ascites fluid did coincide with the late increase in dipeptidase activity of the blood plasma of the same tumour-bearing mice (Ottoson and Sylvén, 1959). Maximum activity figures (in the presence of the dipeptidase inhibitor, previously mentioned) at the end of the growth period were equivalent to about 1·8 $\mu$l. NaOH, which is more than three times the corresponding plasma level. The recorded extracellular activity constitutes from 0·5 to 4 per cent of the total activity contained per $\mu$l. ascites inclusive of the tumour cells.

FIG. 4.—The pH-activity curve of the acid proteinases of washed ascites tumour cells pooled from three mice on the fifth day of tumour growth. Substrate urea-denatured haemoglobin ; cysteine added. Extinctions expressed per $10^6$ cells.

In the case of the proteinases active in the pH range of 2 to 6 the conditions are more complicated. In normal mouse plasma there are at least two (possibly three) proteinases, which show optimum activity against urea-denatured haemoglobin at pH 3·5 (3·5 enzyme) and 4·5 (4·5 enzyme) (Ottoson and Sylvén 1959; Fig. 3). The first one is probably a pepsin-like enzyme (Mirsky *et al.*, 1952), while part of the second one is expected to be of cathepsin nature. The acid proteinases of lymph have not been studied. It may further be added that the extracellular fluid obtained in diluted form from washings of the peritoneal cavity of normal mice also contains two proteinase activity peaks of about equal magnitude at pH 3·5 and 4·5 against urea-denatured haemoglobin (unpublished data). The shape of this pH-activity curve is rather similar to that of blood plasma, but distinctly different from that of the tumour ascites fluid (Fig. 5) mentioned below. In the interstitial fluid of solid mouse tumour transplants two proteinase peaks have also been observed at about pH 3·8 and another at 4·5–4·7 (Sylvén, unpublished data). The pH-dependence of the ascites tumour cell proteinases is shown in Table I and Fig. 4, which demonstrate that the pH 3·5 enzyme is lacking, while, instead, enzymes of the cathepsin group with optimum activity around pH 4·5 are present. On the whole, the cathepsin activity is low in the tumour cells (Malmgren, Sylvén and Révész, 1955 ; Sylvén and Malmgren, 1957). The appearance of some pH 3·5 proteinase in the tumour cell homogenates at the end of the growth period (Table I) is considered to be caused by an admixture of proteins from the ascites fluid not removed by our saline washing.

FIG. 5.—The pH-activity curve of acid proteinases contained in fresh cell-free ascites fluid. Conditions the same as in Fig. 3. Data from three pooled samples of five days old (×——×——×) and twelve days old (●- - - -●) ascites tumours.

TABLE I.—*Serial Data on the Total Dipeptidase and Proteinase Activity of Ascites Tumour Cells*

Each figure obtained on materials pooled from 3 inbred male mice.

| Age of tumour (in days) | Dipeptidase activity (µl. 0·1 M NaOH per $10^4$ cells | Proteinase activity, Extinction increase per $10^6$ cells: Cysteine added, pH 3·5 E | Cysteine added, pH 4·5 E | Cysteine not added, pH 3·5 E | Cysteine not added, pH 4·5 E |
|---|---|---|---|---|---|
| 4 | — | 0·15 | 0·30 | 0·02 | 0·32 |
| 5 | — | 0·00 | 0·94 | — | — |
| 7 | 0·7 | 0·00 | 0·84 | 0·07 | 0·70 |
| 9 | 2·9 | 0·00 | 2·10 | 0·14 | 2·16 |
| 12 | 2·5 | 0·09 | 0·72 | 0·15 | 0·64 |
| 16 | 2·0 | 0·14 | 1·46 | 0·23 | 1·60 |

The ascites tumour fluid, on the other hand, shows certain changes in the pH distribution curve as compared with that of normal mouse blood plasma. During the early phase of tumour growth the pH 1·8–3·5 enzymes are depressed in activity or almost lacking (Fig. 5 and 6), while a 4·5 enzyme of cathepsin-type (thermoand acid-labile) is distinguished. In the course of the later stages of tumour growth the pH distribution gradually changes, more of the pH 3·5 enzyme appears, and further the ratio between the extinction maxima at 3·5 and 4·5 is quite

Fig. 6.—The observed variations in the acid proteinase activity of fresh cell-free ascites fluid at the pH optima 3·5 and 4·5. Each figure obtained from three pooled samples. Activities expressed per µl. fluid (6, A) as well as per µg. protein content (6, B).

different as compared with that in the blood plasma. Fig. 6, A and B, demonstrate the continuous increase in both activities in the later course of ascites tumour growth. The pH 3·5 enzyme in the ascites fluid does not reach the corresponding plasma level of tumour-bearing mice at the end of tumour growth (Ottoson and Sylvén, 1959).

TABLE II.—*Serial Data on the Dipeptidase and Proteinase Activity per μl. Ascites Fluid Pooled from* 3 *Inbred Male Mice*

| Age of tumour (in days) | Dipeptidase activity (μl. 0·1 M NaOH per μl. fluid) | Proteinase activity, Extinction increase per μl. fluid: Cysteine added, pH 3·5 E | Cysteine added, pH 4·5 E | Cysteine not added, pH 3·5 E | Cysteine not added, pH 4·5 E |
|---|---|---|---|---|---|
| 4 | 0·53 | 0·01 | 0·12 | 0·01 | 0·08 |
| 5 | — | 0·00 | 0·32 | — | — |
| 7 | 0·43 | 0·01 | 0·18 | 0·03 | 0·14 |
| 9 | 0·31 | 0·07 | 0·58 | 0·08 | 0·52 |
| 12 | 0·74 | 0·14 | 0·36 | 0·15 | 0·30 |
| 16 | 1·8 | 0·17 | 0·48 | 0·22 | 0·54 |

TABLE III.—*Serial Data on the Dipeptidase and Proteinase Activity per Protein Content in Ascites Fluid Pooled from* 3 *Inbred Male Mice*

| Age of tumour (in days) | Per cent protein content | Dipeptidase activity (μl. 0·1 M NaOH per mg. protein) | Proteinase activity, Extinction increase per mg. protein: Cysteine added, pH 3·5 E | Cysteine added, pH 4·5 E | Cysteine not added, pH 3·5 E | Cysteine not added, pH 4·5 E |
|---|---|---|---|---|---|---|
| 4 | 4·7 | 5·4 | 0·2 | 3·0 | 0·3 | 1·8 |
| 5 | 3·9 | — | 0·0 | 11·0 | — | — |
| 7 | 2·9 | 14·7 | 0·5 | 6·2 | 1·1 | 4·6 |
| 9 | 3·4 | 9·2 | 2·3 | 17·2 | 2·5 | 15·8 |
| 12 | 2·8 | 44·6 | 4·8 | 13·0 | 5·2 | 10·8 |
| 16 | 2·5 | 72·0 | 6·7 | 19·2 | 8·5 | 21·0 |

So far all activities have been expressed per μl. ascites fluid and plasma. The observed increases in enzymic activity are still more marked when correlated to the protein content of the compartments (Table III).

### DISCUSSION

The observed enzymic data illustrate the net results of a complex series of transport and permeability events *in vivo*, which seem difficult to distinguish in detail. Since the enzymes are unstable and are possibly subject to different degrees of dilution and inactivation in all the tissue compartments concerned the conclusions are necessarily vague, and have to be supported by other more direct and independent data. The discussion will mainly be based on the enzymic activities per unit volume as is usual in problems involving transport stable markers. Additional information may be obtained by comparing the enzymic activities on a per-protein basis.

It is further assumed that there is a rapid exchange of water and a slower exchange of protein constituents between the ascites fluid and blood mainly via the lymphatic pathways (Courtice and Steinbeck, 1950 ; Prentice, Siri and Joiner, 1952 ; Abdou, Reinhardt and Tarver, 1952 ; McKee *et al.*, 1952 ; Berson and Yalow, 1954 ; and Schoenberger *et al.*, 1956). It is further presupposed that the ascites fluid originates from the blood serum (Straube, 1958), but also that certain ionic and enzymic contributions may appear from the interstitial fluid of the peritoneal walls as well as from the intracellular compartment of the peritoneal cells, normal as well as tumorous ones.

With reference to the exchange of proteins between the blood and ascites fluid present data seem to justify the conclusion that there is a " restricted diffusion " of globulins into the peritoneal cavity leading to the observed shift in the A/G ratio. This might indicate a certain " sieving-effect " on the part of the capillary membrane.

FIG. 7.—Comparison between the enzymal activities expressed per $\mu$l. fluid in the fluid compartments under question. The plasma data (Ottoson and Sylvén, 1959) are obtained on blood samples from the same mice as used in this report. The data on normal peritoneal fluid will be published elsewhere.

The relative enzymic activities of the fluid compartments under question are for comparison compiled in Fig. 7. According to previous concepts it may be tentatively assumed that the labile extracellular dipeptidase activity is associated with a relatively small protein molecule of a size close to the albumins, while the pH 4·5 proteinase activity most likely resides in a larger molecule possibly of the size of the globulins.

*The dipeptidase concentration*

In the course of the ascites tumour growth the plasma concentration of total dipeptidase activity increased to about twice the normal level (Ottoson and Sylvén, 1959). In the same mice the peptidase activity of the ascites fluid at first, before the 12th day after inoculation, had a similar concentration (Table II), but then rose to a maximum level of about three times that of the corresponding blood plasma activity, and twice that of the normal intraperitoneal fluid (Fig. 7).

The ascites tumour cells had a very high content of peptidases. The results can be explained by the assumption that peptidases are diffusing from the tumour cells into the ascitic fluid and then transported to the blood. The relative balance between the ascites fluid and blood plasma concentration during the first part of tumour growth might signify that the exchange is more rapid during this time (cf. Berson and Yalow, 1954 ; Straube, 1958) while possibly a relative retention of peptidases becomes more evident later on. Independent *in vitro* data (Sylvén, unpublished results) clearly show that the tumour cells are releasing peptidases at a high rate.

*The concentration of acid proteinases*

The proteinase pattern presents a complicated picture. During the first part of the ascites tumour growth most of the acid proteinase activity was abolished both in the blood plasma (Ottoson and Sylvén, 1959) and in the ascites fluid (Fig. 7). It is suggested that this may be due to an increased concentration of acid serum mucoproteins acting as proteinase inhibitiors (cf. above). The pH 3·5 enzyme certainly has some source other than the ascites tumour cells, since the latter do not contain this enzyme (Fig 4). The enzyme on the other hand is present in normal peritoneal fluid (unpublished results), although its actual activity level there is lower than in normal mouse plasma. It is possible that the pH 3·5 enzyme is derived from the stomach mucosa according to Mirsky *et al.* (1952). The present data do not give reliable information as to the actual concentrations of this enzyme due to the apparent presence of an inhibitor, and therefore, further discussion is postponed.

The pH 4·5 proteinase is present in the ascites tumour cells (Fig. 4). Also this enzyme seems to be influenced to some extent by the proteinase inhibitor in the ascites fluid during the first 5 to 7 days of tumour growth. In some series of mice the pH 4·5 enzyme shows a moderate activity in the ascites fluid at the 5th to 7th day after inoculation (Fig. 5). The activity then increases to a high level in the blood plasma as well as in the ascites fluid, although the activity in the latter compartment remains somewhat higher. On the assumption that the inhibitor concentration is of the same magnitude in both compartments, it appears probable that a transport of this enzyme occurs from the cells via the ascites fluid to the blood.

The fate of the peptidases and proteinases possibly transferred to the blood stream is not known ; a certain degree of inactivation and/or excretion is assumed to occur. The peptidases are for instance extremely sensitive, and may as well as the proteinases become excreted similar to its pepsin counterpart. The relative ratio in plasma between the extinctions of the pH 3·5 and the 4·5 proteinase activity leaves no indication as to the fate of the pH 4·5 plasma enzyme during the later part of tumour growth.

It will be noted that the observed differences in enzymic activities per volume would become increased if the data are recalculated per protein content of the different compartments (Table III). Since this may not be fully justified with a view to the transport problems involved, the authors have omitted to consider this set of data in detail.

The present results do not allow us to consider to what extent a selective retention of enzymes might contribute to the observed increases of enzymic activity in the ascites compartment. The determinations are only an indication of the actual activities. Since uncontrolled factors are involved and in particular the presence of a proteinase inhibitor is assumed, the observed activity data do not represent the total enzyme contents, and cannot serve as indicators of the amount of blood proteins filtering through from the serum. Present data are therefore only circumstantial evidence in favour of the view that tumour cells, as well as normal cells, release enzymes to different extents into the extracellular fluid.

The reported *in vivo* experiments, however, are insufficient to explain the factors and environmental conditions responsible for the marked enzymic increase at the end of the ascites tumour growth. It is not known whether this increase is associated with an increased rate of cell death on the part of the tumour cells. Ács and Straub (1954) described a very marked increase in the oncentration of potassium ions in the ascites tumour fluid. This can also be interpreted as an indication of a release of intracellular K ions into the extracellular space. The increase in the K ion concentration was noted already very early during tumour growth, and did not seem to have a close correlation with the frequency of cell death.

The report illustrates the difficulties involved in the interpretation of data derived under *in vivo* conditions, where large differences in enzymic activity are required before significant results can be obtained on a per-volume basis.

### SUMMARY

Quantitative assays were performed of the dipeptidase activity, against alanylglycine, and proteinase activity between pH 3 to 6, using urea-denatured haemoglobin as a substrate, in the cell-free ascites fluid of inbred male mice inoculated with a standard dose of the hyperdiploid Ehrlich ascites tumour. Comparison was made with previous serial assays, performed in the same mice, on the corresponding activities of blood plasma.

The quantitative growth characteristics of this ascites tumour line are described. The protein concentration of the cell-free ascites fluid descreased to a figure of about 2·5 per cent at the end of tumour growth. The changes in both the absolute and relative distribution of albumins and globulins in the blood plasma and ascites fluid of tumour-bearing mice were determined by means of electrophoresis.

The dipeptidase activity of the ascites fluid showed a continuous increase in the course of tumour growth, and reached shortly before the animals died a level of about three times that observed in the corresponding blood plasma. The pH 3·5 and 4·5 proteinases, normally present in blood plasma, were also demonstrable in the normal extracellular peritoneal fluid. The pH 3·5 proteinase activity was almost abolished in the early ascites fluid as well as in the plasma of the same mice. The pH 4·5 activity was simultaneously somewhat depressed. The pH 3·5 proteinase activity later on reappeared in both compartments, but only reached a level of about half the activity of the corresponding blood plasma. The pH 4·5 proteinase activity is similar to the intracellular cathepsins. This reached a high level in the ascites fluid constituting its main proteinase in the acid pH range. During the same time this activity was increased to twice the normal level in the blood plasma.

The conclusion is reached that, most likely, the increase in dipeptidase and pH 4·5 proteinase activity in the ascites fluid originate by release of enzymal proteins from intraperitoneal cells. Other independent data to be reported elsewhere support the view that most of the released enzymes have leaked out from the tumour cells.

The results are so far only valid in the case of ascites tumours; the plasma activities in mice with solid transplanted tumours remain to be studied.

The authors are grateful to Professor Torsten Theorell, University of Uppsala, for his encouragement and criticism. The investigation has been supported by institutional grants from the Swedish Cancer Society, the King Gustaf V's Jubilee Fund, and the Jane Coffin Childs Memorial Fund for Medical Research, which are all gratefully acknowledged.

### REFERENCES

ABDOU, I. A., REINHARDT, W. O. AND TARVER, H.—(1952) *J. biol. Chem.*, **194**, 15.
ÁCS, G. AND STRAUB, F. B.—(1954) *Acta Physiol. hung.*, **6**, 253.
BAYREUTHER, K.—(1952) *Z. Naturf.*, **7**, 554.
BENSON, J. A., KIM, K. S. AND BOLLMANN, J. L.—(1955) *Amer. J. Physiol.*, **182**, 217.
BERSON, S. A. AND YALOW, R. S.—(1954) *J. clin. invest.*, **33**, 377.
BOSCH, L.—(1958) *Biochim. biophys. Acta*, **30**, 444.
BRAUN-FALCO, O.—(1957) *J. Histo- Cytochem.*, **5**, 94.
BURSTONE, M. S.—(1956) *J. nat. Cancer Inst.*, **10**, 1149.
CHRISTENSEN, H. N. AND RIGGS, T. R.—(1952) *J. biol. Chem.*, **194**, 57.
COURTICE, F. C. AND STEINBECK, A. W.—(1950) *Aust. J. exp. Biol. med. Sci.*, **28**, 161.
DOYLE, W. L.—(1955) *J. Biophys. Biochim. Cytol.*, **1**, 221.
FEINSTEIN, R. N. AND BALLIN, J. C.—(1953) *Cancer Res.*, **13**, 780.
FLEISHER, G. A. AND BUTT, H. R.—(1953) *J. clin. Invest.*, **32**, 674.
FRUTON, J. S.—(1946) *J. biol. Chem.*, **166**, 721.
GRASSMANN, W. AND HEYDE, W.—(1929) *Z. physiol. Chem.*, **183**, 32.
HAUSCHKA, T. S., GRINNEL, S. T., RÉVÉSZ, L. AND KLEIN, G.—(1957) *J. nat. Cancer Inst.*, **19**, 13.
ISHINO, K.—(1935/38) *Arb. anat. Inst. Univ. Kyoto*, 5–7, 68.
KUN, E., TALALAY, P. AND WILLIAMS-ASHMAN, H. G.—(1951) *Cancer Res.*, **11**, 855.
LEDOUX, L. AND REVELL, S. H.—(1955) *Biochim. biophys. Acta*, **18**, 416.
LIBENSON, L. AND JENA, M.—(1957) *Cancer*, **10**, 1004.
MALMGREN, H., SYLVÉN, B. AND RÉVÉSZ, L.—(1955) *Brit. J. Cancer*, **9**, 473.
MCKEE, F. W., YULIE, C. L., LAMSON, B. G. AND WHIPPLE, G. H.—(1952) *J. exp. Med.*, **95**, 161.
MIRSKY, A., FUTTERMAN, P., KAPLAN, S. AND BROH-KAHN, R. H.—(1952) *J. Lab clin. Med.*, **40**, 17.
OSATO, S.—(1921) *Tohoku J. exp. Med.*, **2**, 514.
OTTOSON, R. AND SYLVÉN, B.—(1959) *Arch. Biochem. Biophys.* (in press).
PRENTICE, T. C., SIRI, W. AND JOINER, E. E.—(1952) *Amer. J. Med.*, **13**, 668.
RÉVÉSZ, L. AND KLEIN, G.—(1954) *J. nat. Cancer Inst.*, **15**, 253.
SCHADE, A. L.—(1953) *Biochim. biophys. Acta*, **12**, 163.
SCHOENBERGER, J. A., KROLL, G., ECKERT, E. L. AND KARK, R. M.—(1956) *J. Lab. clin. Med.*, **47**, 227.
STRAUBE, R. L.—(1958) *Cancer Res.*, **18**, 57.
SYLVÉN, B.—(1945) *Acta radiol. Stockh.*, Suppl. 59, " Ester Sulphuric Acids of High Molecular Weight and Mast Cells in Mesenchymal Tumors.—(1954) *Acta Un. int. Cancr.*, **10**, 169.

SYLVÉN, B. AND MALMGREN, H.—(1957) *Acta radiol. Stockh.*, Suppl. 154, " The Histological Distribution of Proteinase and Peptidase Activity in Solid Tumor Transplants"
WARBURG, O. AND CHRISTIAN, W.—(1943) *Biochem. Z.*, **314**, 399.
WU, R. AND RACKER, E.—(1958) *Fed. Proc.*, **17**, 239.
YOFFEY, J. M. AND COURTICE, F. C.—(1956) " Lymphatics, Lymph and Lymphoid Tissue ". 2nd Ed. London (E. Arnold Publ. Ltd.).